# Multi-omics analyses reveal the effects of layerage and grafting on flavonoid synthesis and accumulation in *Citrus reticulata* ‘Chachi’

**DOI:** 10.1093/hr/uhaf177

**Published:** 2025-07-07

**Authors:** Jianmu Su, Mingmin Jiang, Huimin Pan, Weitao Zhou, Xueyan Cai, Yukun Wang, Wei Liu, Desen Wang, Mei Bai, Hong Wu

**Affiliations:** Centre for Medicinal Plant Research, College of Life Sciences, South China Agricultural University, 483 Wushan Road, Tianhe District, Guangzhou 510642, China; College of Biology and Agriculture, Shaoguan University, 288 Daxue Road, Xinshao Town, Zhenjiang District, Shaoguan 512005, China; Centre for Medicinal Plant Research, College of Life Sciences, South China Agricultural University, 483 Wushan Road, Tianhe District, Guangzhou 510642, China; Centre for Medicinal Plant Research, College of Life Sciences, South China Agricultural University, 483 Wushan Road, Tianhe District, Guangzhou 510642, China; Centre for Medicinal Plant Research, College of Life Sciences, South China Agricultural University, 483 Wushan Road, Tianhe District, Guangzhou 510642, China; Centre for Medicinal Plant Research, College of Life Sciences, South China Agricultural University, 483 Wushan Road, Tianhe District, Guangzhou 510642, China; College of Biology and Agriculture, Shaoguan University, 288 Daxue Road, Xinshao Town, Zhenjiang District, Shaoguan 512005, China; Centre for Medicinal Plant Research, College of Life Sciences, South China Agricultural University, 483 Wushan Road, Tianhe District, Guangzhou 510642, China; National Key Laboratory of Green Pesticide, Department of Entomology, South China Agricultural University, 483 Wushan Road, Tianhe District, Guangzhou 510642, China; Centre for Medicinal Plant Research, College of Life Sciences, South China Agricultural University, 483 Wushan Road, Tianhe District, Guangzhou 510642, China; Guangdong Key Laboratory for Innovative Development and Utilization of Forest Plant Germplasm, South China Agricultural University, 483 Wushan Road, Tianhe District, Guangzhou 510642, China; Centre for Medicinal Plant Research, College of Life Sciences, South China Agricultural University, 483 Wushan Road, Tianhe District, Guangzhou 510642, China; Guangdong Key Laboratory for Innovative Development and Utilization of Forest Plant Germplasm, South China Agricultural University, 483 Wushan Road, Tianhe District, Guangzhou 510642, China

## Abstract

Guangdong Citri Reticulatae Pericarpium from the dry and mature peel of *Citrus reticulata* ‘Chachi’ (CRC) is a well-known medicinal and food material in Asia. The main propagation methods of CRC are layerage and grafting. It is generally considered that the quality of CRC from layerage is superior to that obtained from plants propagated by grafting. Nevertheless, the effects of layerage and grafting on the biosynthesis of flavonoid (main bioactive ingredients) in the peel of CRC remain unknown. Here, metabolomic analyses revealed the effects of layerage, self-grafting, and heterografting (*Citrus limonia* as rootstock) on flavonoid biosynthesis in CRC from two main harvesting periods, CRCV (Citri Reticulatae Chachiensis Viride) and CRCR (Citri Reticulatae Chachiensis Reddish). Compared with CRCR, CRCV exhibited a higher content of flavonoids. Grafting CRC onto *C. limonia* exhibited a higher content of hesperidin, nobiletin, tangeretin, narirutin, demethylnobiletin, and sinensetin than layerage and self-grafting. This increase can be attributed to the upregulation of genes involved in flavonoid synthesis. Further, the transcription factor *CrcMYBF1* was identified within the gene coexpression network and is confirmed to be significantly induced by methyl jasmonate (MeJA) and upregulate the expression of *Crc1,6RhaT* through interacting with its promoter region, thereby boosting the biosynthesis and accumulation of hesperidin. In summary, our findings provide mechanistic insights into the coordinated regulation of hesperidin biosynthesis via MeJA-inducing *CrcMYBF1* in CRC. Our study is expected to provide a theoretical basis for CRC propagation and cultivation.

## Introduction

Guangdong Citri Reticulatae Pericarpium refers to the dry and mature peel of *Citrus reticulata* ‘Chachi’ (CRC) [1] and is a well-known medicinal and food material with a long history of utilization in Southeast Asia and East Asia [2, 3]. As the main bioactive components of Citri Reticulatae Pericarpium, flavonoids exhibit antioxidant [4], anticancer [5, 6], anti-inflammatory [7], antimicrobial [8, 9], antiviral [10], and attenuation of metabolic syndrome [11] properties and have great clinical value [12].

Flavonoids are significant for both human health and plant vitality, facilitating growth and development while aiding in the resistance to biotic and abiotic stresses. Flavonoids enhance the resistance of rice against *Pyricularia oryzae* and play a crucial role in protecting plant cell from UV damage [13, 14]. Hesperidin, a main flavonoid in Citri Reticulatae Pericarpium, has been proven to have antibacterial effects and as the marker components of Citri Reticulatae Pericarpium recorded in the Chinese Pharmacopeia [1]. The hesperidin content was relatively high at the early developmental stage and showed a gradually decreasing trend at the fruit ripe developmental stage [15]. The biosynthesis of hesperidin commences with cinnamic acid and involves a series of enzymatic steps catalysis by phenylalanine ammonia-lyase (PAL), 4-coumarate-CoA ligase (4CL), chalcone synthase (CHS), chalcone isomerase (CHI), flavone 3′- hydroxylase (F3'H), flavone 4’-*O*-methyltransferase (F4’OMT), hesperidin 7-*O*-glucoside transferase (7-*O*-GlcT), and CRC 1,6-rhamnosyltransferase (Crc1,6-RhaT). Previous studies revealed that 1,6-rhamnosyltransferase was the synthase of hesperidin [15–17]. However, the regulation of the accumulation of hesperidin remains unclear.

Propagation methods such as layerage and grafting are common in citrus plants. Existing studies revealed that different rootstocks affect citrus water transport [18], physiological metabolism [19, 20], yield and fruit quality [21, 22], stress resistance [23], antioxidant activity [24], and secondary metabolites [22, 25, 26]. Grafting has been shown to have a significant impact not only on the secondary metabolites of citrus fruits, but also on those of other species, such as melon and grape [27–29]. Especially, jasmonate plays a crucial role in the accumulation of second metabolites in heterografting chrysanthemum [30]. Rootstock-derived miRNAs can be transported upward through the phloem [31]. Previous studies implied that mRNAs may be transferred from the rootstock to the scion, further regulate the accumulation of secondary metabolites in the scion [32–34]. These studies suggest that grafting not only affects the growth and development of plants, but also influences the synthesis and accumulation of secondary metabolites in plants. Currently, the three common propagation methods used in production are layerage, self-grafting, and heterografting. The rootstock mainly used in heterografting is *Citrus limonia* Osbeck. Layerage exhibits weak resistance to diseases and insect pests, while self-grafting and heterografting exhibits strong resistance to diseases and insect pests [26]. However, it remains unclear what effects grafting has on the biosynthesis and accumulation of flavonoids in the peel of CRC. Using multi-omics to study the regulatory mechanisms of the synthesis and accumulation of bioactive components in medicinal plants is a convenient and reliable method [35].

In this study, metabolomics and transcriptomics were employed to investigate the differences in flavonoid content and associated gene expression in the pericarp of CRC from different propagation methods at two important harvest periods, CRC Viride (CRCV, ‘Qing Pi’ in Chinese; around July) and CRC Reddish (CRCR, ‘Wei Hong Pi’ in Chinese; around November). And further to uncover the difference of flavonoids between different rootstocks and the mechanisms of transcriptional regulatory in hesperidin synthesis and accumulation. Our findings provide new insights into the effects of grafting on citrus fruit quality and medicinal ingredients.

## Results

### Qualitative and quantitative analysis of the metabolites

**Figure 1:**
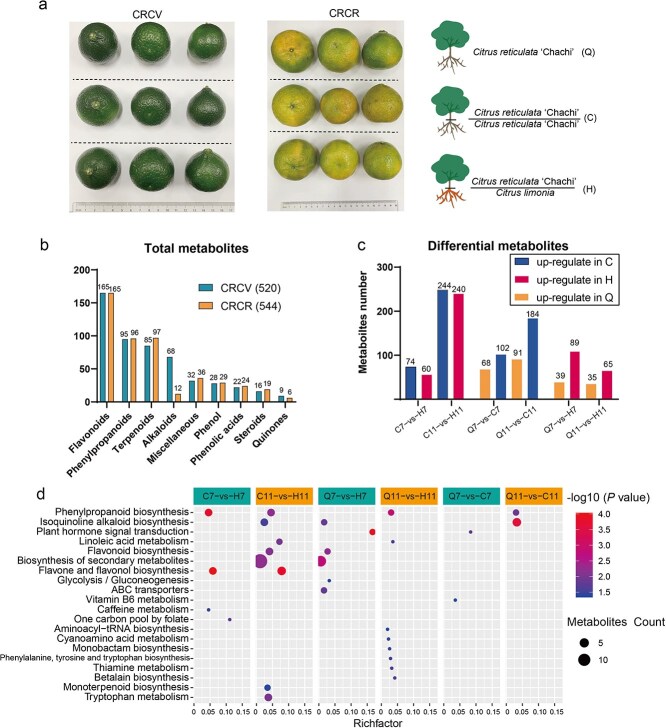
Sample information and metabolite features. (a) The grafting design and citrus fruit phenotypes of *C. reticulata* ‘Chachi’ from different propagation methods. The plant materials included three types, layerage (graft-free, named group Q), heterografted (*C. limonia* Osbeck as rootstock, named group H), and self-grafted (named group C) plants. (b) Total metabolites identified in all the samples. Green and orange represent the CRCV and CRCR periods, respectively. (c) Differential content of metabolites in each comparison group. Metabolites with a fold change ≥1.2 or ≤0.83 and a *P-*value ≤ 0.05 were differentially accumulated metabolites. Each group included three biological replicates. (d) Pathway enrichment of the differential content of metabolites in each comparison group. Pathways with *P-*value ≤ 0.05 are shown. The node size represents the number of differentially abundant metabolites within the pathway. ‘Metabolite count’ represents the number of metabolites enriched in each pathway. ‘Rich factor’ represents the percentage of total differentially abundant metabolites.

To investigate the effect of different propagation methods on the medicinal components of *C. reticulata* ‘Chachi’ (CRC), a nontarget metabolomics analysis was performed on the pericarp of CRC. A total of 520 and 544 metabolites were identified in the CRCV and CRCR periods, respectively (Fig. 1b, Supplementary Fig. 1). Among these, flavonoids and flavonoid glycosides were the most abundant class in all the samples, accounting for 165 metabolites (Fig. 1b). This was followed by phenylpropane, with 95 and 96 metabolites identified in samples from CRCV and CRCR, respectively (Fig. 1b). Terpenoids are important essential oils in the peel of CRC, with 85 and 97 metabolites in samples from CRCV and CRCR, respectively (Fig. 1b). Notably, the number of alkaloids in the CRCV samples was significantly higher than that in the CRCR samples (68 and 12, respectively). These results demonstrate that citrus peels contain a diverse range of secondary metabolites, primarily flavonoids, followed by phenylpropane derivatives and terpenoids (Fig. 1b).

To compare the metabolic differences between groups, the identified metabolite data were subjected to fold change analysis and *t*-tests. In the CRCV samples, 134, 170, and 128 differentially accumulated metabolites were identified in the C7 (self-grafted citrus; samples collected at July) vs H7 (graft onto *C. limonia* Osbeck; samples collected at July), Q7 (graft-free citrus; samples collected at July) vs C7, and Q7 vs H7 comparisons, respectively. Among these, 74 and 102 metabolites were upregulated in the C7 group compared to the H7 and Q7 groups, respectively, while 60 and 114 were upregulated in the H7 group compared to the C7 and Q7 groups, respectively. Additionally, 68 and 39 metabolites were upregulated in the Q7 group compared to the C7 and H7 groups, respectively (Fig. 1c). In the CRCR, 484, 275 and 100 differentially accumulated metabolites were identified in the comparisons of C11 (self-grafted citrus; samples collected at November) vs H11 (graft onto *C. limonia* Osbeck; samples collected at November), Q11 (graft-free citrus; samples collected at November) vs C11, and Q11 vs H11, respectively (Fig. 1c). The C11 group showed 244 and 184 upregulated metabolites in comparison to the H11 and Q11 groups, respectively. The H11 group demonstrated 240 and 65 upregulated metabolites compared with the C11 and Q11 groups, respectively (Fig. 1c). Additionally, the Q11 group displayed 91 and 35 upregulated metabolites in comparison to the C11 and H11 groups, respectively (Fig. 1c). Interestingly, in both the CRCV and CRCR samples, the Q11 vs H11 comparison had the fewest differentially accumulated metabolites, suggesting that the metabolic content of the Q11 and H11 groups was more similar. Overall, the differences in metabolite content among pericarp samples collected from different propagation methods during the CRCR period were greater than those among samples collected during the CRCV period.

To investigate the metabolic pathways linked to the differentially abundant metabolites, an enrichment analysis of the metabolic pathways was performed. The findings revealed that 20 metabolic pathways exhibited enrichment with differentially abundant metabolites across all comparison groups (Fig. 1d). Notably, the phenylpropanoid biosynthetic pathway was enriched in four comparison groups (C7 vs H7, C11 vs H11, Q11 vs H11, Q11 vs C11), while the isoquinoline alkaloid biosynthetic pathway was enriched in three comparison groups (C11 vs H11, Q7 vs H7, Q11 vs C11). Furthermore, the plant hormone signal transduction pathway was enriched in two comparison groups (Q7 vs H7, Q7 vs C7). Additionally, significant enrichment was observed in the linoleic acid metabolism, flavonoid biosynthesis, and flavonoid and flavonol biosynthetic pathways (Fig. 1d).

In the CRCV samples, differentially abundant metabolites from the C7 vs H7 comparison were enriched in four metabolic pathways, including the phenylpropanoid biosynthetic pathway, flavonoid and flavonol biosynthetic pathway, caffeine metabolism, and one carbon pool by folate (Fig. 1d). Differentially abundant metabolites from the Q7 vs H7 comparison were enriched in six metabolic pathways, with the secondary metabolite pathways having the most differentially abundant metabolites, followed by isoquinoline alkaloid biosynthesis, plant hormone signal transduction, and flavonoid biosynthetic pathways (Fig. 1d). Differentially abundant metabolites from the Q7 vs C7 comparison were enriched in two metabolic pathways, the plant hormone signal transduction pathway, and the vitamin B6 metabolism pathway (Fig. 1d). In the CRCR samples, differentially abundant metabolites from the C11 vs H11 comparison were enriched in eight metabolic pathways, including phenylpropanoid biosynthesis and flavonoid and flavonol biosynthetic pathways (Fig. 1d). Differentially abundant metabolites from the Q11 vs H11 comparison were enriched in eight metabolic pathways, with the phenylpropanoid synthesis pathway having the most differentially abundant metabolites. Differentially abundant metabolites from the Q11 vs C11 comparison were significantly enriched in two metabolic pathways, phenylpropanoid biosynthetic and isoquinoline alkaloid biosynthetic pathways (Fig. 1d).

**Figure 2:**
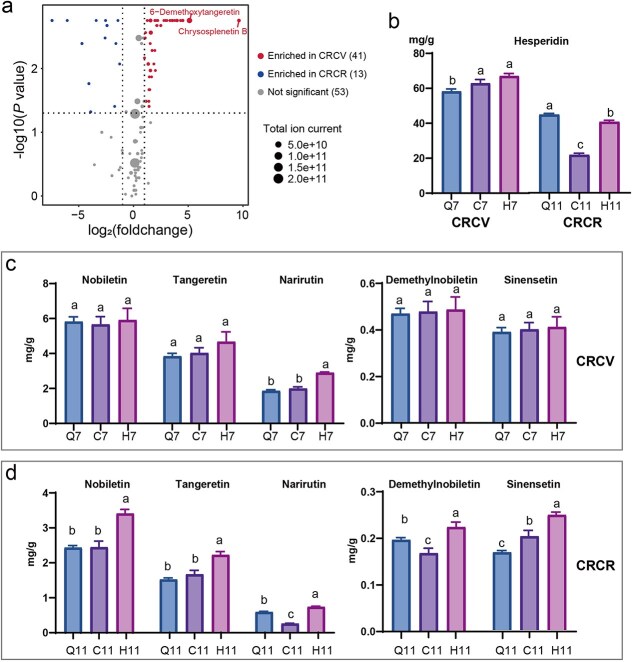
Flavonoid contents of the three groups at the CRCV and CRCR. (a) Comparison of the differential flavonoids in the CRCV and CRCR groups, each group included three biological replicates. (b) The content of hesperidin in the pericarp of *C. reticulata* ‘Chachi’ plants grafted with different propagation methods at the CRCV. Different lowercase letters above the bars indicate the statistically significant differences using Duncan’s multiple comparison test. (c, d) The content of flavonoids in the pericarp of *C. reticulata* ‘Chachi’ from different propagation methods during the CRCV and CRCR periods.

Flavonoids are the most important secondary metabolites in the peel of CRC. A comparison of CRCV and CRCR revealed that 41 flavonoids were enriched in CRCV, and 13 flavonoids were enriched in CRCR (Fig. 2a). Among them, the levels of 6-demethoxytangeretin and chrysosplenetin B exhibited the greatest differences between the CRCV and CRCR (Fig. 2a). These results implied that the content of most flavonoids was enriched at the CRCV (Fig. 2b). To further investigate the differences in several important flavonoids among the different groups, we performed absolute quantification analysis of hesperidin, nobiletin, tangeretin, narirutin, demethylnobiletin, and sinensetin. The results showed that in the CRCV samples, only the contents of narirutin and hesperidin exhibited different among the different groups, while the other four flavonoids showed no significant differences (Fig. 2b and c). The content of hesperidin was highest in the H7 group, reaching 67.05 mg/g, which was significantly higher than that in the Q7 group (58.21 mg/g). The content of hesperidin in the C7 group was 62.90 mg/g, which was also significantly higher than that in the Q7 group. There was no significant difference in the hesperidin content between the H7 and C7 groups (Fig. 2b). The content of narirutin showed the same trend as that of hesperidin, with the highest content in the H7 group, followed by the C7 group, and the lowest in the Q7 group; the contents were 2.90, 2.00, and 1.86 mg/g, respectively. The H7 group had a significantly higher content than did the C7 and Q7 groups (Fig. 2c). In the CRCR samples, all six flavonoids showed significant differences among the different groups. The Q11 group had the highest content of hesperidin, followed by the H11 group, and the lowest was in the C11 group, with contents of 40.63, 36.86, and 19.66 mg/g, respectively (Fig. 2b). The trends in the hesperidin, nobiletin, and tangeretin contents were consistent among the three groups, with the highest occurring in the H11 group, followed by the C11 group, and the lowest occurring in the Q11 group (Fig. 2b and d). The contents of sinensetin and demethylnobiletin showed similar trends, with the highest in the H11 group, followed by the Q11 group, and the lowest in the C11 group (Fig. 2d). With respect to the CRCR samples, all five flavonoids except hesperidin were most abundant in the H11 group. In summary, CRC grafting onto *C. limonia* Osbeck can increase the content of flavonoids in the pericarp of CRC.

### Identification of differentially expressed genes and pathway enrichment

**Figure 3:**
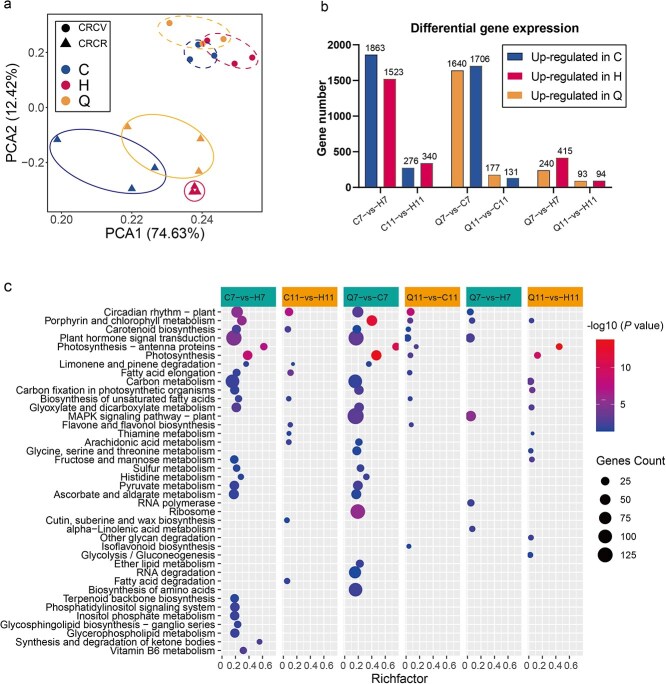
Gene expression patterns and differential gene enrichment. (a) Principal component analysis of gene expression in all samples. Dots represent the CRCV, and triangles represent the CRCR. (b) Number of genes upregulated in each comparison group. Blue, red, and orange represent genes upregulated in the C, H, and Q groups, respectively. (c) Pathway enrichment of DEGs in each comparison group. Pathways with *P-*value ≤ 0.05 are shown. The node size represents the number of DEGs within the pathway. ‘Gene count’ is the number of genes enriched in a pathway. ‘Rich factor’ is the percentage of total DEGs in the given pathway term.

Differences in metabolites originate from differences in gene expression. Exploring the gene expression of different propagation methods can further elucidate the molecular mechanisms of metabolite biosynthesis. We further conducted transcriptomic sequencing analysis on all the samples to identify the gene expression patterns in the pericarp of *C. reticulata* ‘Chachi’ (CRC) from the different propagations. A total of 771 million clean paired-end reads were obtained from the RNA-seq dataset for 18 samples. Each sample yielded an average of 6.43 Gb of data, with Q20 > 97% clean reads (Supplementary Table 1). The principal component analysis (PCA) results showed that the samples from the CRCV and CRCR were clearly separated, indicating significant differences in gene expression between samples from different periods (Fig. 3a). In the CRCV samples, the differential expression genes (DEGs) in the C7 vs H7, Q7 vs C7, and Q7 vs H7 comparison groups were 3386, 3346, and 655, respectively, with a total of 74 DEGs shared among the three comparison groups (Fig. 3b, Supplementary Fig. 2). In the CRCR samples, the DEGs in the Q11 vs C11, Q11 vs H11, and C11 vs H11 comparison groups were 308, 187, and 616, respectively, with three DEGs shared among the three comparison groups (Fig. 3b, Supplementary Fig. 2). The comparison between C7 and H7 revealed the highest number of differentially expressed genes (DEGs), with 1863 genes upregulated in C7 and 1523 genes upregulated in H7 (Fig. 3b). In the comparison of Q7 vs C7, a substantial number of DEGs were identified, with 1640 genes upregulated in Q7 and 1706 genes upregulated in C7 (Fig. 3b). Conversely, in the comparison of Q7 vs H7, a lower number of DEGs were observed, with 240 genes upregulated in Q7 and 415 genes upregulated in H7 (Fig. 3b). In the pericarp from the CRCR period, all compared groups showed a lower number of DEGs compared to the CRCV period. These results indicated that there was a large difference in gene expression between the pericarp of grafted and layerage CRC at the CRCV.

The pathway enrichment analysis of DEGs were performed and revealed that these genes are involved mainly in plant circadian rhythms, porphyrin and chlorophyll metabolism, carotenoid biosynthesis, plant hormone signal transduction, photosynthesis-antenna proteins, and photosynthesis pathways (Fig. 3c). The differentially expressed genes in the C11 vs H11 comparison group were enriched in the biosynthetic pathways of flavones and flavonols. The DEGs in the Q11 vs C11 comparison group were also involved in the biosynthesis of flavone and flavonol, and the isoflavonoid biosynthetic pathways (Fig. 3c). Five pathways were simultaneously enriched in the metabolic pathways of differentially expressed genes and in the pathways of differentially enriched metabolites, including plant hormone signal transduction, flavone and flavonol synthesis, glycolysis/gluconeogenesis, vitamin B6 metabolism, and thiamine metabolism (Fig. 3c). These enriched metabolic pathways of differentially expressed genes may collectively influence the differences in metabolite content in the pericarp of *C. reticulata* ‘Chachi’ from different propagation methods.

### Metabolome and transcriptome conjoint analysis to identify genes associated with flavonoid biosynthesis

**Figure 4:**
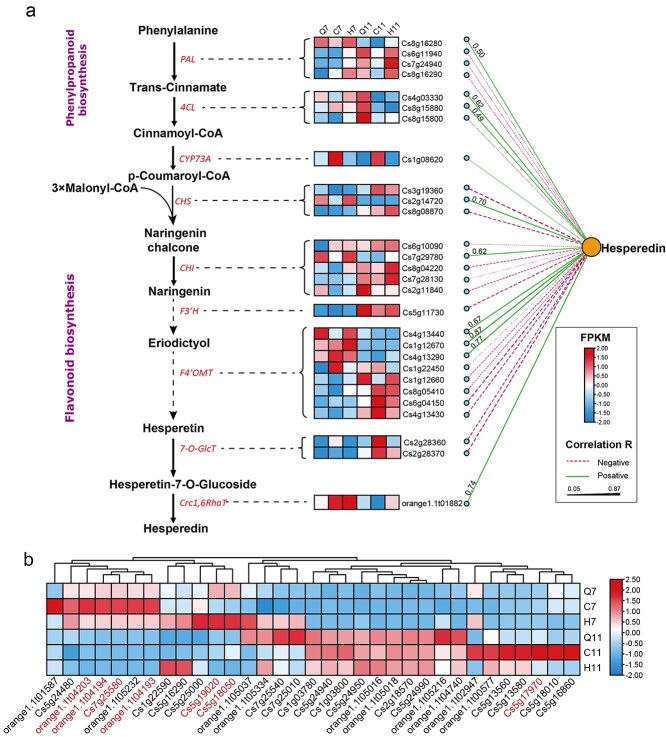
Expression of genes involved in the biosynthesis of hesperidin. The FPKM values of the genes were normalized. PAL, phenylalanine ammonia-lyase; 4CL, 4-coumarate-CoA ligase; CYP73A, trans-cinnamate 4-monooxygenase; CHS, chalcone synthase; CHI, chalcone isomerase; F3′H, flavone 3′- hydroxylase; F4′OMT, flavone 4′-*O*-methyltransferase; 7-*O*-GlcT, hesperetin-7-*O*-glucosyltransferase; Crc1,6RhaT, *C. reticulata* ‘Chachi’ hesperetin-1,6-rhamnosyltransferase. The Correlation coefficients between genes and hesperidin contents are shown beside the line. The solid and dashed lines represent positive and negative relationships, respectively. (b) Gene expression of Caffeic acid 3-*O*-methyltransferase. Genes in red represent gene have been demonstrated to improve the content of nobiletin and tangeretin.

Hesperidin is the flavonoid glycoside with the highest content in the pericarp of *C. reticulata* ‘Chachi’ (CRC). Correlation analysis between the expression levels of these genes and the content of hesperidin revealed that, except for 7-*O*-GlcT*,* the expression of the other eight genes was positively correlated with the biosynthesis of hesperidin (Supplementary Table 2). In particular, F4’OMT *Cs1g12670* (*r* = 0.87), *Cs4g13290* (*r* = 0.77), Crc1,6-RhaT *orange1.1 t01882* (*r* = 0.74), and CHS *Cs2g14720* (*r* = 0.70) exhibited strong correlations with the content of hesperidin (Fig. 4a). This finding suggested that these three genes may play a crucial role in the biosynthesis of hesperidin. Additionally, caffeic acid O-methyltransferase (COMT) plays an important role in the accumulation of Polymethoxyflavones (PMFs) in citrus. Thirty-three O-methyltransferases (COMTs)were identified in present study. Among them, 11 COMTs were upregulated in CRCV, in which seven COMTs were upregulated in C7 and four COMTs were upregulated in H7 (Fig. 4b). Twenty-three COMTs were upregulated in CRCR, in which 13 COMTs exhibited significant difference and the number of genes upregulated in Q11, C11, and H7 were four, seven, and two, respectively (Fig. 4b). Seven genes, *orange1.1 t04203*, *orange1.1 t04194*, *Cs7g25580*, *orange1.1 t04193*, *Cs5g19020*, *Cs5g18050*, and *Cs5g17970*, have been demonstrated to improve the content of nobiletin and tangeretin. In which six COMTs were upregulated in CRCV and one COMT was upregulated in CRCR (Fig. 4b). The quantitative reverse transcription polymerase chain reaction (qRT-PCR) results of seven COMT genes and *Crc1,6-RhaT* further confirmed that the RNA-seq data was credible (Supplementary Fig. 3). The upregulated COMTs in C7, H7, C11, and H11 may boost the accumulation of nobiletin and tangeretin.

To further explore the genes that influenced the accumulation of flavonoid compounds in the pericarp of CRC, metabolomic and transcriptomic coexpression analyses were performed. The results showed that all genes were clustered into 33 modules (Supplementary Fig. 4), and each module contained 33–2862 genes (Supplementary Fig. 5). Correlation analysis between the gene modules and the contents of six major flavonoid compounds revealed that the orange module contained 75 genes, with correlation coefficients (R) of 0.79 and 0.73 for narirutin and hesperidin, respectively (Supplementary Fig. 5). The dark gray module contained 96 genes, with R values of 0.68 and 0.67 for narirutin and hesperidin, respectively (Supplementary Fig. 5). The green module included 1539 genes, and the R values of the modules correlated with narirutin and hesperidin were 0.74 and 0.73, respectively (Supplementary Fig. 5). These results suggest that genes in orange, dark gray, and green modules exhibited a significant impact on the biosynthesis and accumulation of hesperidin.

**Figure 5:**
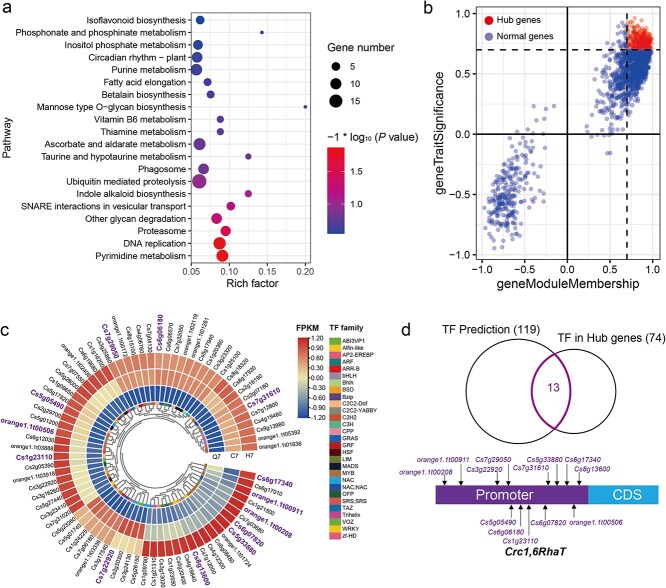
Identification of hub genes and *cis*-elements involved in *Crc1,6RhaT* biosynthesis. (a) Pathway enrichment of genes in the green module. The pathway term with *P-*value ≤ 0.05 are shown. Node size represents the number of genes within the pathway. ‘Gene number’ represents the number of genes enriched in a pathway. ‘Rich factor’ represents the percentage of total genes in the given pathway term. (b) Hub gene identification. Hub genes were identified by screening genes that correlated with hesperidin in the green module base on gene module membership ≥0.7 and gene trait significance ≥0.7. (c) The FPKMs of the TFs in the green module. FPKM values were centered and scaled in the row. TFs are classed on the right. (d) TF-binding site prediction of the *Crc1,6RhaT* promoter. The Venn diagram showed the intersection of TF prediction and TF in hub genes. The binding site of 13 candidate TFs were sorted at *Crc1,6RhaT* promoter.

Since the green module contains the greatest number of genes among these three modules, we first focused our attention on the green module. In the green module, a total of 1274 genes were positively correlated with hesperidin, and these genes were mainly enriched in pathways such as pyrimidine metabolism, DNA repair, proteasome, other polysaccharide degradation, SNARE interactions in vesicular transport, indole alkaloid biosynthesis, circadian rhythm-plant, and isoflavonoid biosynthesis (Fig. 5a). We further identified 297 hub genes related to hesperidin based on a gene module membership (GMM) ≥0.7 and a gene trait (hesperidin) significance (GTS) ≥0.7 (Fig. 5b). Among these 297 genes, 74 transcription factors (TFs) were identified and belonged to 27 TF families, including 15 MYBs, 7 C2CE-Dofs, 7 bHLHs, and 5 NACs (Fig. 5c). To investigate the TFs regulating the *Crc1,6RhaT* gene among the hub genes, we first predicted the TF-binding sites in the promoter of the hesperidin biosynthetic gene *Crc1,6RhaT*. The results showed that the *Crc1,6RhaT* promoter contains a total of 205 TF-binding sites, which belong to 119 TF families (Supplementary Table 3). These 119 TF families shared 13 TFs with the 74 TFs among the hub genes, such as *Cs5g33880* (named *CrcMYBF1*), *orange1.1 t00208* (C2C2-YABBY), *orange1.1 t00911* (MYB), *Cs3g22920* (bHLH), and *Cs7g29050* (C2C2-Dof) (Fig. 5d).

### CrcMYBF1 directly binds to the *Crc1,6RhaT* promoter region and activates the *Crc1,6RhaT* transcription

**Figure 6:**
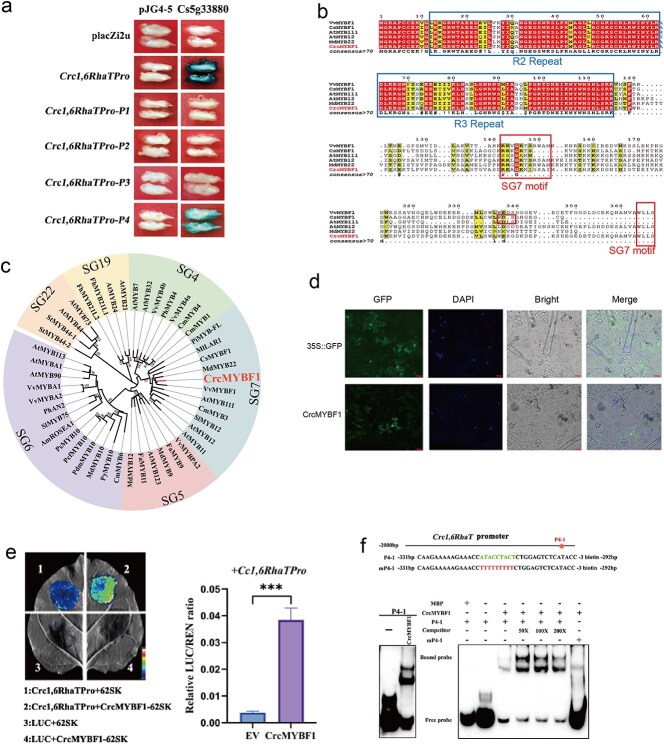
*CrcMYBF1* binds to P4-1 *cis*-element and activates the *Crc1,6RhaT* expression. (a) Y1H analysis of CrcMYBF1 binding to the *Crc1,6RhaT* promoter (*Crc1,6RhaTPro*). AD-empty and placZi2u were used as negative controls. *Crc1,6RhaTPro-P1* to *Crc1,6RhaTPro-P4* represents four parts of promoter. (b) Sequence alignment of CrcMYBF1 and AtMYB12, AtMYB111, and VvMYBF1 (*Vitis vinifera* 'Shiraz' R2R3-MYB TF VvMYBF1) proteins. The lines below the sequences represent the R2 and R3 conserved domains, respectively, the box represents the SG7 motif. (c) Phylogenetic relationship of CrcMYBF1 and other plant R2R3-MYB proteins. CrcMYBF1 is marked in red and belongs to the SG7 subgroup of R2R3-MYB. Other plant R2R3-MYB proteins can be retrieved in Uniprot. (d) Subcellular localization of CrcMYBF1 in *N. benthamiana* (DAPI was used for nuclear staining), empty eGFP vector was used as a control, bars = 20 μm. (e) The activity of *Crc1,6RhaT* promoters fused to the LUC reporter was determined by Dual-LUC assays. Empty pGreenII-62SK vector and *Crc1,6RhaT*-pGreenII-0800 vector were measured with LUC/REN set as negative control values, and SE values were calculated with three biological replicates. Statistical significance was determined by *t*-test (‘***’ represents *P* < 0.0001). (f) Schematic diagram of *Crc1,6RhaT* promoter and probes used for EMSA. The binding site of CrcMYBF1 is indicated in green, and the mutated base is indicated in red. P4-1 represents the probe, which is the predicted binding site of *CrcMYBF1*, and mP4-1 represents the mutant probe. The competitive probe is tested with 50-, 100-, and 200-fold unlabeled biotin. The empty vector MBP-tagged protein replaces the CrcMYBF1 protein as a negative control.

A yeast one-hybrid (Y1H) assay was used to further verify the interaction of 13 TFs and *Crc1,6RhaT* promoter. The results showed that only *Cs5g33880* (named *CrcMYBF1*) could directly bind to the *Crc1,6RhaT* promoter, whereas the remaining 12 TFs did not demonstrate this binding capability (Fig. 6a, Supplementary Fig. 6). Further, the expression of *CrcMYBF1* was compared between layerage or grafting citrus. The results showed that, for sample harvest at July, the expression of *CrcMYBF1* in self-grafting and graft onto *C. limonia* was higher than layerage (Supplementary Fig. 7). For sample harvest at November, the expression of CrcMYBF1 in graft onto *C. limonia* was higher than self-grafting and layerage (Supplementary Fig. 7). The expression of *Crc1,6RhaT* showed a consistent pattern with *CrcMYBF1* and exhibited high correlation with *CrcMYBF1* in the coexpression network (Supplementary Fig. 7). Sequence alignment analysis on CrcMYBF1 revealed that the N-terminus of CrcMYBF1 contains a conserved R2R3-MYB domain and its C-terminus contains SG7 and SG7-2 motifs (Fig. 6b). To analyze the phylogenetic relationship of CrcMYBF1, a phylogenetic tree was constructed with CrcMYBF1 and 45 R2R3-MYB in other plants. The results showed that CrcMYBF1 formed a branch with VvMYBF1, MdMYB22, and CsMYBF1, indicating that CrcMYBF1 belongs to the SG7 MYB family (Fig. 6c). Subcellular localization showed that CrcMYBF1 was located in the nucleus (Fig. 6d). Furthermore, dual-luciferase reporter assays conducted in tobacco leaves showed that CrcMYBF1 can directly bind to the promoter of *Crc1,6RhaT* and activate the expression of *Crc1,6RhaT* (Fig. 6e). Additionally, the prediction of the PlantTFDB (https://planttfdb.gao-lab.org/) indicated that CrcMYBF1 specifically recognizes the 5'-ACC(A/T)A(A/C)-3′ motif. Analysis of the *Crc1,6RhaT* promoter revealed a potential *cis*-acting element denoted as P4-1 (Fig. 6f). Further, electrophoretic mobility shift assay (EMSA) experiments were performed using MBP-CrcMYBF1 fusion protein. The results showed that CrcMYBF1 specifically binds to the P4-1 motif of the *Crc1,6RhaT* promoter (Fig. 6f). These results implied that CrcMYBF1 directly binds to the promoter of *Crc1,6RhaT* at P4-1 *cis*-acting element.

### CrcMYBF1 promotes *Crc1,6RhaT* expression and hesperidin biosynthesis

**Figure 7:**
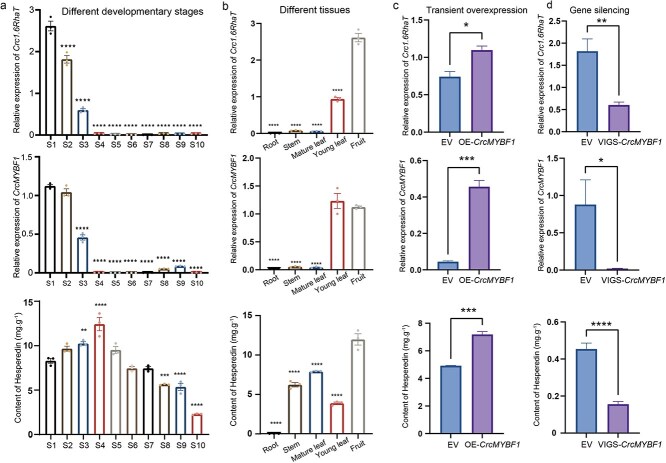
Overexpression and RNAi-induced silencing of *CrcMYBF1* in citrus. (a) The expression of *CrcMYBF1* and *Crc1,6RhaT* and content of hesperidin from difference development stages of citrus. The content of hesperidin was determined using fresh peel without freeze-drying. S1–S10 represent 35, 65, 95, 110, 130, 150, 170, 200, 230, and 260 days after flowering, respectively. Differential analysis was performed using S1 as the control group. (b) The expression of *CrcMYBF1* and *Crc1,6RhaT* and content of hesperidin from difference tissues of citrus. Differential analysis of different development stages and different tissues was performed using S1 and fruit as the control group, respectively. (c) Overexpression of *CrcMYBF1* in the peel of citrus. The transcription levels of *CrcMYBF1*, *Crc1,6RhaT* and the content of hesperidin were detected after 3 days of treatment. The EV group was the empty vector pBI121, OE-*CrcMYBF1* represents Overexpression of *CrcMYBF1*. (d) The transcription levels of *CrcMYBF1*, *Crc1,6RhaT*, and content of hesperidin after virus-induced *CrcMYBF1* gene silencing. The EV group was the empty vector pBI121, VIGS-*CrcMYBF1* represents virus-induced gene silencing of *CrcMYBF1*. All data represent the means SDs (*n* ≥ 3 as indicated in the figure; ‘*’ represents *P* < 0.01; ‘**’ represents *P* < 0.001; ‘***’ represents *P* < 0.0001; ‘****’ represents *P* < 0.00001 Student’s *t*-test).

To confirm the relationship between CrcMYBF1 and the accumulation of hesperidin in the peel of *C. reticulata* ‘Chachi’ (CRC), the gene expression of *CrcMYBF1* and *Crc1.6RhaT* in the fruit development stage and different tissues was analyzed by qRT-PCR. The results showed that *CrcMYBF1* and *Crc1,6RhaT* exhibited the same expression pattern during the fruit development of CRC. Specifically, the expression level of *CrcMYBF1* and *Cc1,6RhaT* was the highest in the S1 stage, and gradually decreased with the development of the fruit, very low expression level was found in the S4–10 stages (Fig. 7a). The content of hesperidin exhibited a pattern of initial increase with the highest level observed at the S4 stage, followed by a decrease throughout the fruit development process (Fig. 6a). In different tissues, *Crc1,6RhaT* demonstrated highest expression in the peel, whereas *CrcMYBF1* showed highest expression in young leaves and peel. Concurrently, the peel contained the highest abundance of hesperidin (Fig. 7b). These results speculate that *CrcMYBF1* activates the expression of *Crc1,6RhaT* and thus participates in hesperidin synthesis.

To verify the role of *CrcMYBF1* in the accumulation of hesperidin in citrus, transient overexpression of *CrcMYBF1* was first carried out in citrus fruit peels. The results indicated a significant increase in the expression of *CrcMYBF1* and *Crc1,6RhaT*, as well as in the content of hesperidin, in comparison to the control group (Fig. 7c). Further, we used virus-induced gene silencing to interfere with the expression of *CrcMYBF1* in CRC seedlings. The data revealed a significant reduction in the expression levels of *CrcMYBF1* and *Crc1,6RhaT* compared to the control in citrus seedlings, resulting in a significant decline in hesperidin content (Fig. 7d). Taken together, these results directly demonstrate that *CrcMYBF1* acts as a positive regulator of hesperidin biosynthesis by activating the transcription of *Crc1,6RhaT*.

**Figure 8:**
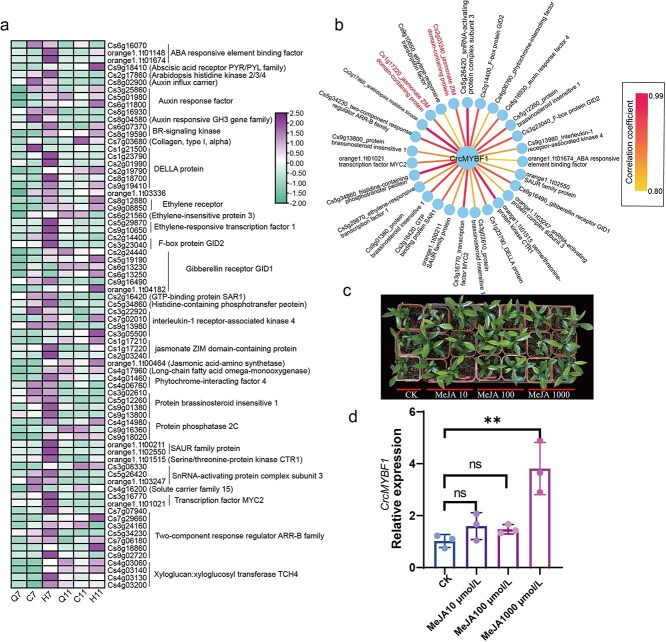
Gene networks of plant hormone signal transduction and relative expression of *CrcMYBF1.* (a) Gene expression heatmap of genes in plant hormone signal transduction pathway. The group designations Q, C, and H correspond to layerage, self-grafted, and heterografted citrus. ‘7’ and ‘11’ indicate the harvest times of July and November. Gene expression levels have been scaled. (b) The correlation network between *CrcMYBF1* and genes in plant hormone signal transduction pathway. (c) One-year-old seedlings of *C. reticulata* ‘Chachi’ treated with MeJA. (d) Relative expression of CrcMYBF1 after the treatment of MeJA with 10, 100, and 1000 μmol/l. CK represents ultrapure water treatment.

In order to further explore the factors affecting the upregulated expression of *CrcMYBF1*, we further explored the gene expression patterns in citrus under three different grafting methods. The transcriptome data analysis revealed that differential genes mainly enriched in plant hormone signal transduction pathway (Fig. 3c), which implied hormone signal may play a crucial role in the differential expression of *CrcMYBF1*. Additionally, the gene expression in plant hormone signal transduction showed that 74 genes were upregulated in H (graft onto *C. limonia* Osbeck) or C (self-grafted citrus) group (Fig. 8a), compared with layerage. In them, *Cs3g23040* (F-box protein), *Cs9g16490* (gibberellin receptor GID1), *orange1.1 t01021* (TF MYC2), *Cs4g03200* (xyloglucan: xyloglucosyl transferase TCH4), and *Cs1g17220* (jasmonate ZIM domain-containing protein) exhibited higher expression in H and C than that in layerage (Fig. 8a). A correlation network was constructed basing on the Spearman correlation coefficient between *CrcMYBF1* and 74 hormone-associated genes. As a result, *CrcMYBF1* were correlated significantly with 27 genes, such as *Cs5g26420*, *Cs2g17860*, *Cs5g34860*, *orange1.1 t01515*, *Cs3g16770*, *Cs1g17220*, and *Cs2g03240* (Fig. 8b). In them, *Cs1g17220* and *Cs2g03240* annotated as jasmonate ZIM domain-containing protein and exhibited highly correlation coefficient with 0.91 and 0.81, respectively (Fig. 8b). These results implied that the expression of *CrcMYBF1* may be regulated by jasmonate. To prove our hypothesis, we evaluated the impact of methyl jasmonate (MeJA) treatment on the expression of *CrcMYBF1.* The results indicated that the expression level of *CrcMYBF1* was significantly increased after 24 h of treatment with 1000 μmol/l MeJA, compared with those of untreated controls. In contrast, no significant differences in expression level were observed between the untreated controls and the treatments with 100 μmol/l and 10 μmol/l MeJA. (Fig. 8c). These results indicated that MeJA boosts expression of the TF *CrcMYBF1*. Our findings provide mechanistic insights into the coordinated regulation of hesperidin biosynthesis via JA inducing *CrcMYBF1*.

## Discussion

As a traditional Chinese medicinal material, dried peel of *C. reticulata* ‘Chachi’ (CRC) has been shown to exhibit numerous therapeutic functions [1]. Peel of CRC harvested at different times exhibited varied medicinal values [11]. By comparison of layerage and grafting citrus plants, our results revealed that the contents of major flavonoids, including hesperidin, nobiletin, tangeretin, sinensetin, narirutin, and demethylnobiletin in CRCV of CRC harvested in July were higher than those in CRCR of CRC harvested in November. Our study supported that the content of flavonoids in CRC peel decreases with delayed harvest time [36], and CRCV and CRCR of CRC should be used for different clinical treatment.

Propagation methods such as layerage and grafting are common in citrus plants. Different methods of propagation have significant impact on the growth, development, physiology, and biochemical characteristics of plants [37]. For example, propagating watermelon plants through grafting with pumpkin rootstocks can increase plant height, stem thickness, and leaf area and improve root system morphology and root vitality [38]. Grafting can also improve the plant morphology and physiological traits of cucumbers, stabilizing vegetable crop yield and quality [39,40]. In grapevines, more anthocyanin was found in self-grafted samples than in nongrafted samples, due to the anthocyanin biosynthesis-related genes upregulated in self-grafted samples [29]. Rootstocks can have varying effects on the growth vigor, photosynthesis, yield, fruit quality, carbohydrate content, stress resistance, and synthesis of secondary metabolites of citrus plants [25,41]. Mineral nutrient absorption and accumulation in citrus plants, especially the biosynthesis and accumulation of terpenoids, can also be influenced by rootstock [42]. In this study, we found that the content of flavonoids and the expression of genes involved in flavonoid biosynthesis in fruit peel of CRC were influenced by different propagation methods, and the impact effect varied in the CRCV and CRCR of CRC. In the CRCV period, only hesperidin and narirutin exhibited differences among the three groups. However, in the CRCR period, the contents of all six major flavonoids significantly differed among the three groups, indicating that the effect of different propagation methods on flavonoids content of CRC peel manly occurred during the CRCR period. Tan *et al*. [26] reported that grafting for CRC had no significant effect on the content of hesperidin, nobiletin, tangeretin, total flavonoids, and total polymethoxylated flavones, while more researches expressed the view that rootstocks do have an impact on sugar content in citrus pulp [43,44]. Our study on a wider scale including three different propagation methods and two harvesting period indicated that grafting for CRC had significant effect on six major flavonoids in CRCR period. Heterografting can better improve the resistance of CRC and improve the flavonoid content of PCRC; using *C. limonia* as rootstocks in production may be an efficient and high-quality method for propagating CRC.

Hesperidin, nobiletin, and tangeretin are important bioactive ingredients for the clinical treatment of Citri Reticulatae Pericarpium, and are new efficacy indices in the new version of Chinese pharmacopeia [1]. Our results revealed that grafting boosts the accumulation of hesperidin, nobiletin, and tangeretin. Recently, COMTs were reported to play a curial role in the accumulation of nobiletin and tangeretin [45]. We found that more COMTs were upregulated in two grafting citrus groups than that in layerage group, providing additional support for the increased content of PMFs nobiletin and tangeretin in grafting citrus. Hesperidin, being the predominant flavonoid in Peels of CRC, is pivotal in shaping the medicinal attributes of Peels of CRC [46]. *Crc1,6RhaT* has been reported to catalyze the conversion of hesperetin-7-*O*-glucoside to hesperidin [15]. Multiple copies of *1,6RhaT* were identified in various citrus germplasms, whereas only type A alleles encode a functional protein [17]. In the present study, only one *Crc1,6RhaT* was found and the expression of *Crc1,6RhaT* was higher in the CRCV than in the CRCR, consistent with the content changes of hesperidin. Pericarp from fruit of citrus plants grafted with *C. limonia* as rootstock exhibited the highest expression of *Crc1,6RhaT* compared with that in self-grafted and layerage plants, also consistent with the content changes of hesperidin. Taken together, these results indicated that the upregulation of *COMTs* and *Crc1,6RhaT* boosts the accumulation of nobiletin, tangeretin, and hesperidin in grafting citrus.

TFs play prominent roles in regulating gene expression [47]. In recent years, studies have identified TFs that regulate the expression of genes involved in plant flavonoid biosynthesis [48–50]. MYB TFs are a big TF family in plants and play a crucial role in the biosynthesis flavonoid biosynthesis [48,51,52]. By analyzing expression regulation of 13 candidate TFs related to the expression of *Crc1,6RhaT* and accumulation of hesperidin, *Cs5g33880* (*CrcMYBF1*) was identified. Y1H, dual luciferase (Dual-LUC), and EMSA assays demonstrated that *CrcMYBF1* could directly bind to the *Crc1,6RhaT* promoter and activated the expression of *Crc1,6RhaT.* Previous studies have demonstrated that many members of t MYB families play important roles in regulating flavonoid biosynthesis, such as PpMYB114, MsMYB62, FaMYB5, and MdMYB9 [53–56, 57]. However, the function of *CrcMYBF1* was different with precious MYB TFs, which bind to the *cis*-elements of promoters of chalcone synthase (*CHS*), flavanone carboxylase (*F3H*), and flavonol synthase 1 (*FLS1*) to regulate flavonoid metabolism [48]. Further, overexpression and RNAi-induced silencing of *CrcMYBF1* demonstrated that *CrcMYBF1* promoted the accumulation of hesperidin. These findings confirm that variations in hesperidin content among different rootstocks are attributable to the differential expression of *CrcMYBF1* and *Crc1,6RhaT*. MYB was triggered by jasmonate signaling and further regulated the enhancement of secondary metabolites [58]. Previous studies reported that MeJA response elements are often found in promoter regions of MYB genes, indicating that MYB genes are regulated by MeJA [50, 59]. In the present study, we found genes annotated as jasmonate ZIM domain-containing protein had a strong correlation with *CrcMYBF1*. Further exogenous application of MeJA revealed that MeJA directly induced the upregulation of *CrcMYBF1*. By integrating our findings with existing literature, we inferred that the MeJA–*CrcMYBF1* regulatory pathway is universal in citrus plants. Our results confirmed that the rich accumulation of hesperidin in grafted citrus via MeJA induced high expression of *CrcMYBF1.*

Taken together, grafting CRC boosts the accumulation of hesperidin and four PMFs—nobiletin, tangeretin, desmethylnoiletin, and sinensetin—particularly with the highest levels observed in citrus grafted onto *C. limonia*. In the grafting CRC, the upregulated TF *CrcMYBF1* induced by MeJA binds to the promoter of *Crc1,6RhaT*, thereby promoting the expression of *Crc1,6RhaT*, and ultimately leading to the accumulation of more plentiful hesperidin. These suggest that grafting CRC, especially *C. limonia*, should be used as the rootstock of CRC for high quality of Citri Reticulatae Pericarpium production to improve the contents of hesperidin and PMFs.

## Conclusions

*Citrus reticulata* ‘Chachi’ (CRC) grafted onto *C. limonia* boosted the accumulation of main bioactive ingredients flavonoids in their pericarps by high expression of TFs induced by MeJA and of gene-related flavonoids synthesis genes. CRC grafted onto *C. limonia* is beneficial to the production of high-quality Citri Reticulatae Pericarpium and the sustainable development of Citri Reticulatae Pericarpium industries. Additionally, the contents of major flavonoids, including hesperidin, narirutin, nobiletin, tangeretin, sinensetin, and demethylnobiletin in CRCV of CRC were higher than those in CRCR of CRC.

## Materials and methods

### Plant materials

Samples were collected from Shuangshui Town, Xinhui District, Jiangmen City, Guangdong Province, China, at two harvest periods of the CRCV and CRCR (Table 1). Fresh fruits from three different plant propagation types were collected in the same orchard. Three biological replicates were performed for each type at each collection. To ensure the consistency of the samples, four citrus fruits of the same size facing four directions from one tree and mix their peels as one sample. The plant materials included three types: layerage (graft-free, named group C), self-grafted (named group Q), and heterografted (*C. limonia* Osbeck as rootstock, named group H). According to SNP identification [60], all the scions of the three different plant propagation types originated from the same line of *C. reticulata* ‘Chachi’. The pericarp of the fruits was obtained and used for further metabolome analysis and transcriptome analysis.

**Table 1:** Sample information on three different plant propagation types.

Groups	Plant propagation	Rootstock	Collecting time	Periods
Q7	Layerage		10 July	CRCV
C7	Grafting	*C. reticulata* ‘Chachi’	10 July	CRCV
H7	Grafting	*C. limonia* Osbeck	10 July	CRCV
Q11	Layerage		10 November	CRCR
C11	Grafting	*C. reticulata* ‘Chachi’	10 November	CRCR
H11	Grafting	*C. limonia* Osbeck	10 November	CRCR

### Metabolome analysis

The untargeted metabolomics analysis was conducted at BGI Genomics Co., Ltd. (Shenzhen, China). The metabolites were extracted from the pericarp of the fruits (50 mg). A quality control (QC) sample was generated by combining 20 μl of supernatant from each sample to assess the reproducibility and stability of the comprehensive liquid chromatography-mass spectrometry (LC–MS) analysis. The raw data obtained from liquid chromatography-tandem mass spectrometry (LC–MS/MS) were imported into Compound Discoverer 3.1. (Thermo Fisher Scientific, USA) for data processing. Data preprocessing was performed using metaX [61].

Metabolites with a fold change ≥1.2 or ≤0.83 and a *P-*value ≤0.05 were differentially accumulated metabolites (DAMs). The metabolic pathway enrichment analysis of differentially abundant metabolites was carried out based on the KEGG database, and the metabolic pathways with *P-*value ≤0.05 were those with significant enrichment of differentially abundant metabolites.

### Flavonoid extraction and quantification

Flavonoids were extracted and determined according to our previous study [62,63]. The 50-mg freeze-dried CRC peel powder was placed in a 1.5-ml centrifuge tube, and 800 μl of methanol was added, followed by ultrasound in a 4°C water bath for 30 min. The supernatant was centrifuged at 12 000 g for 30 min, filtered through 0.22-μm organic membrane, and then put into a vial for ultra performance liquid chromatography (UPLC) detection. The high-performance liquid chromatography (HPLC) system was an Agilent 1260 HPLC system equipped with a vacuum degasser, a G1316A quadruple pump, a G1315C diode array detector (DAD), and a G1367E autosampler. The mobile phase A was acetonitrile and mobile phase B was 0.1% formic acid at a flow rate of 0.8 ml/min. The column temperature was set at 40°C with an injection volume of 2 ul and a UV detection wavelength of 283 nm. The samples were run with the following gradient program: 0–2 min, 5% acetonitrile; 2–22 min, 5%–95% acetonitrile; 22–27 min, 95% acetonitrile; 27.1–30 min, 5% acetonitrile.

To generate the standard curve, we first prepared different concentration gradients of hesperidin, narirutin, nobiletin, tangeretin, sinensetin, and demethylnobiletin standard solutions. Then, we filtered all the solutions to be loaded onto the machine through a 0.22 μM organic filter membrane into a sample bottle with an inner tube, and then loaded onto the machine for detection. The standard curve of each standard was drawn with concentration as the horizontal axis and peak area as the vertical axis. The regression equation. The absolute content of hesperidin, narirutin, nobiletin, tangeretin, sinensetin, and demethylnobiletin in each sample was calculated.

### RNA extraction, detection, and sequencing

Total RNA was extracted from the peel samples by using a Column Plant RNAout Kit (Cat#: 71203-50; TIANDZ Gene Technology, Beijing, China) according to the manufacturer’s protocol. The quality of the RNA samples was assessed using a Qubit Fluorometer and an Agilent 2100 to confirm their adequacy for RNA sequencing. The mRNA was fragmented with an interrupting buffer, followed by reverse transcription using random N6 primers to synthesize complementary DNA (cDNA) and produce double-stranded DNA. The ligated products were subsequently amplified and denatured to form single strands, which were then circularized with a bridge primer to create a single-stranded circular DNA library. This library was subsequently sequenced on the DNBSEQ platform.

### Gene differential expression analysis

The raw data were filtered to exclude low-quality sequences by SOAPnuke software [64]. Cleaned reads were mapped to the reference genome [65] by HISAT2 [66]. The expression levels of genes and transcripts were calculated by ballgown [67]. The DEseq2 software package was used to detect DEGs using log_2_|foldchage| ≥1 and a Q-value ≤0.05 as parameters [68].

### Conjoint analysis of the metabolome and transcriptome

A gene coexpression network was established utilizing the weighted gene correlation network analysis (WGCNA) package (version 4.0.2) [69] in R. Genes were selected for WGCNA if they were expressed in at least 80% of the samples and exhibited fragments per kilobase of exon per million mapped fragments (FPKM) values ≥1. The analysis parameters included power = 12, minModuleSize = 30, and MEDissThreshold = 0.25. Genes within modules that correlated with flavonoid content were employed to construct coexpressed gene and metabolite networks. Hub genes were identified by filtering for genes exhibiting correlations with flavonoids (GTS ≥ 0.7 and GMM ≥ 0.7). The hub gene coexpression network, along with the resulting gene–gene interactions, was visualized using Gephi (version 0.92) [70].

### Transcription factor-binding site prediction of *Crc1,6RhaT*

The promoter and CDS of *Crc1,6RhaT* were obtained from our previous studies [15,63]. TF-binding site prediction was performed with the web-based tool PlantRegMap (https://plantregmap.gao-lab.org/). The sequence of the promoter was dropped from the web-based tools, and the *P-*value threshold was set to <0.0001.

### Yeast one-hybrid assay

For Y1H assay, the coding regions of *Cs5g33880* were cloned into the pJG4-5 (AD) vector (Takara Bio, USA) at the *EcoRI* and *XhoI* restriction sites to generate the *AD-Cs5g33880* constructs. The promoter fragments of *Crc1,6-RhaT* (2 kb from the ATG site) were amplified and cloned into the pLacZi2μ vector digested with *EcoRI* and *XhoI*, Creating *Crc1,6RhaTp-pLacZi2u* vector [71]. To test the binding of *Cs5g33880-AD* to the promoters of *Crc1,6RhaT*, the *Cs5g33880-AD* and *Crc1,6RhaTp-pLacZi2u* plasmids were cotransformed into the yeast strain *EGY48* and selected on SD/-Ura/-Trp medium (Takara Bio, USA) containing raffinose, galactose, and 5-bromo-4-chloro-3-indolyl-b-D-galactopyranoside (Sangon Biotech, China) for blue color development.

### Subcellular localization

To investigate the subcellular localization of CrcMYBF1, the open reading frame of *CrcMYBF1* was inserted into pCAMBIA1300-eGFP vector. *Agrobacterium* strains carrying GFP fluorescent tags were grown in LB liquid medium containing kanamycin (50ug/ml) and rifampicin (25ug/ml) at 30°C and 200 rpm overnight. The bacterial solution was collected at 6000 rpm for 10 min and resuspended in infection buffer (10 mM MgCl_2_, 10 mM MES, 150uM acetosyringone pH = 5.6) to OD600 = 0.7. The suspension was injected into leaves of *Nicotiana benthamiana* grown for 1 month, and empty pCAMBIA1300-eGFP was used as a blank control. Two days later, the epidermis of the injection area of the tobacco leaves was taken for fluorescence observation using a laser confocal scanning microscope.

### Dual luciferase assays

Firstly, the promoter sequence of *Crc1,6RhaT* was inserted into pGreen II0800 -LUC vector, while *CrcMYBF1* was inserted into pGreen II 62SK vector, and transformed into *Agrobacterium* strain GV3101 (pSoup), respectively. The *Agrobacterium* strains containing the TF-SK recombinant plasmid and the promoter-LUC recombinant plasmid were cultured overnight in LB selection medium. Afterward, the bacterial suspension was collected and resuspended in infection solution to an OD600 of 0.75, followed by mixing the TF with the promoter at a ratio of 10:1 (v/v). Uniformly sized and healthy *N. benthamiana* plants were selected for infection. As a negative control, *Agrobacterium* containing the empty SK vector was mixed with the promoter *Crc1,6RhaT*-LUC and injected. Three days postinfection, samples were taken using a puncher with a diameter of ~2 mm near the injection sites, with three small discs taken from each leaf. The samples were then ground and centrifuged, and the supernatant was used for detection. The Dual-luciferase Reporter Assay System kit (Yesen, China) was employed for the analysis.

### Electrophoretic mobility shift assays

The full-length open reading frame of *CrcMYBF1* was inserted into pMAL-c5X vector to generate maltose-tagged fusion protein, and the plasmid was transformed into *Escherichia coli* Rosetta (DE3) (Weidi, China). The biotin-labeled probe and the cold probe used in the experiment were both labeled using the biotin labeling kit (B.Y.T., China). Prior to the reaction, the upstream and downstream primers were annealed to synthesize double-stranded probes. The biotin-labeled probe and the recombinant protein were then incubated at room temperature for 20 min. After the reaction, 6% native polyacrylamide gel electrophoresis was performed at 120 V for 45 min. Following electrophoresis, the nylon membrane (Thermo, USA) was crosslinked under a UV lamp for 15 min. Finally, chemiluminescent detection was carried out using a chemiluminescent substrate.

### Transient overexpression in citrus fruit peel

The experimental method was modified based on Zhao *et al*. [72]. The full-length coding sequence of *CrcMYBF1* was inserted into the pBI121 vector, and then the constructed vector was transformed into the *Agrobacterium* strain EHA105 (Weidi, China). *Agrobacterium* activation was performed by resuspending the bacteria in infiltration buffer (10 mM MgCl_2_, 100uM acetosyringone, 10 mM MES pH 5.6) to an OD600 = 1. Uniform fruit without mechanical damage or pest infestation were selected. The suspension of cell carrying the target gene and empty vector was injected into peel of the fruit. Subsequently, the fruits were kept in darkness for 24 h and then kept under a 16-h light and 8-h dark photoperiod. Every three fruits were mixed to form one sample and three biological replicates for each sample. Flavonoid content and gene expression were determined 3 days after treatment.

### Virus-mediated gene silencing in citrus seedlings

A 300-bp conserved sequence from the CDS region of *CrcMYBF1* was amplified and inserted into the TRV2 vector. Both TRV1 and the constructed TRV2 vector were then transformed into the *Agrobacterium* strain GV3101. According to Wang *et al*. [73], VIGS was performed by placing *Agrobacterium* liquid carrying *CrcMYBF1*-TRV2 and TRV1 in LB liquid medium, and cultivating overnight at 28°C and 200 rpm. The bacterial liquid was collected, resuspended in infiltration buffer (10 mM MgCl_2_, 100uM acetosyringone, 10 mM MES pH 5.6) to an OD_600_ = 1, and mixed TRV1 with TRV2 or TRV1 with *CrcMYBF1*-TRV2 in a 1:1 volume ratio, then incubated at room temperature for at least 3 h. Citrus seedlings were immersed in the *Agrobacterium* liquid, placed in a vacuum pump, and vacuumed for 15 min. After infiltration, the seedlings were grown in the dark for 2 days, then transferred to a plant growth chamber for 1 month, followed by sample collection for metabolite content detection and gene expression analysis.

### Validation of the RNA sequence data using qRT-PCR

The RNA was extracted and reverse transcribed into cDNA using the PrimeScript RT reagent Kit (TaKaRa, Dalian, China). Quantitative real-time PCR (qRT-PCR) was used to detect the expression levels of the eight genes in flavonoid biosynthesis pathway. Each sample consisted of three biological replicates, and each biological replicates consisted of three technical replicates. The relative expression level was calculated by the double delta Ct. All primers of genes were listed in Supplementary Table 4.

### Hormone-associate genes mining and MeJA treatment

The FPKM of genes upregulated in the C and H groups were extracted and presented in a heatmap. Spearman correlations between *CrcMYBF1* and hormone-associated genes were calculated using the R package *cor.test*. *P-*values were filtered at a threshold of 0.05, and the resulting genes were used to construct a correlation network in Cytoscape (v 3.10.3). For MeJA treatment, 1-year-old seedlings of *C. reticulata* ‘Chachi’ were used and the concentration of MeJA was 10, 100, 1000 μmol/l, and the control group was sprayed with ultrapure water. After 24 h of treatment, the leaves were collected for RNA extraction and qPCR to detect the expression of *CrcMYBF1*. The primers were listed in Supplementary Table 4.

### Statistical analysis

The data collation and analysis were performed with Excel 2020 (Microsoft Corporation, Redmond, WA, USA). Spearman correlations between two variables were calculated by the R package *cor.test*. One-way analysis of variance (ANOVA) and Student’s *t*-test were performed using the R package *t test*. The corresponding statistical graphs were generated using GraphPad Prism (version 8.3.0.538) and the R package ggplot2.

## Supplementary Material

Web_Material_uhaf177

## Data Availability

All raw data were deposited in the GenBank NCBI Short Read Archive under accession number PRJNA03126.
