# Supplementary material for: Multi-omics analyses reveal the effects of layerage and grafting on flavonoid synthesis and accumulation in Citrus reticulata ‘Chachi’
Source: Hortic Res. 2025 Jul 7;12(10):uhaf177. doi: 10.1093/hr/uhaf177 (PMC12532756; doi:10.1093/hr/uhaf177)
Supplement: Web_Material_uhaf177 [file web_material_uhaf177.zip › Supplementery Figures-Rootstocks.docx]

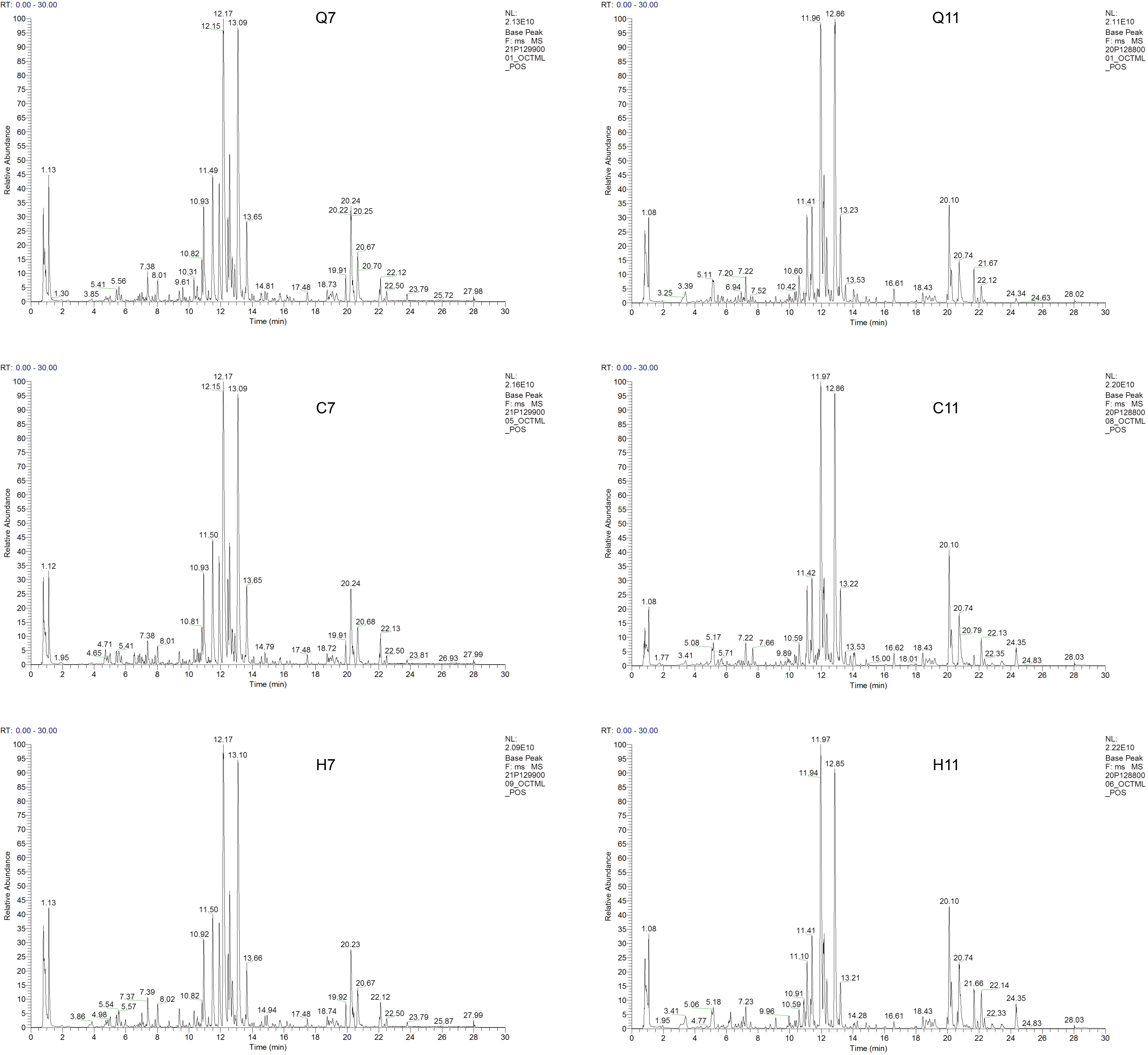


**Figure S1 Multi-peaks chromatograms of metabolites using multiple reactions monitoring**


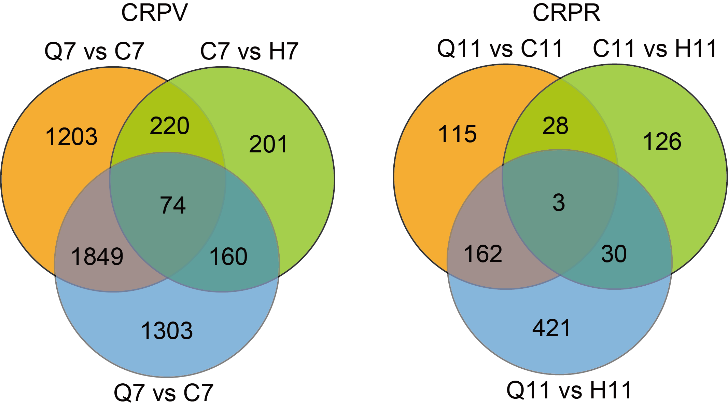


**Figure S2 Differential genes in three comparison groups at CRPV and CRPR**

**
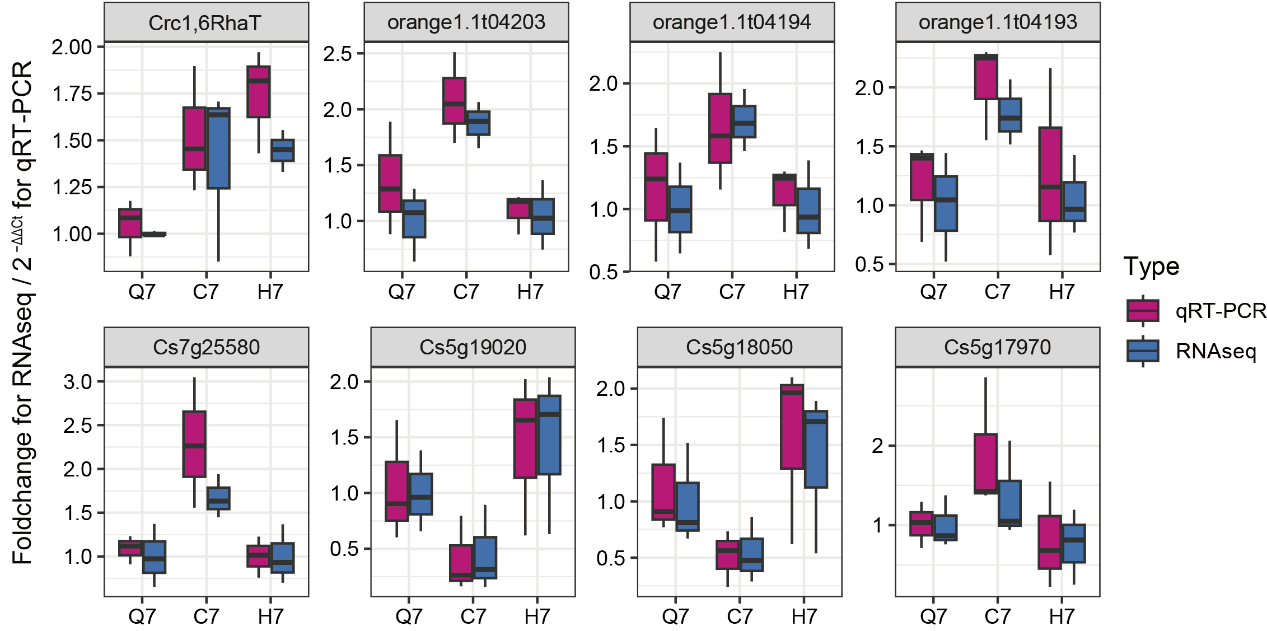
**

**Figure S3 qRT-PCR validation profiles of eight selected genes.** The expression levels of genes in Q7 were used as the reference state, which was set to 1, and fold change values and 2^−ΔΔCt^ were shown for RNAseq and qRT-PCR, respectively.


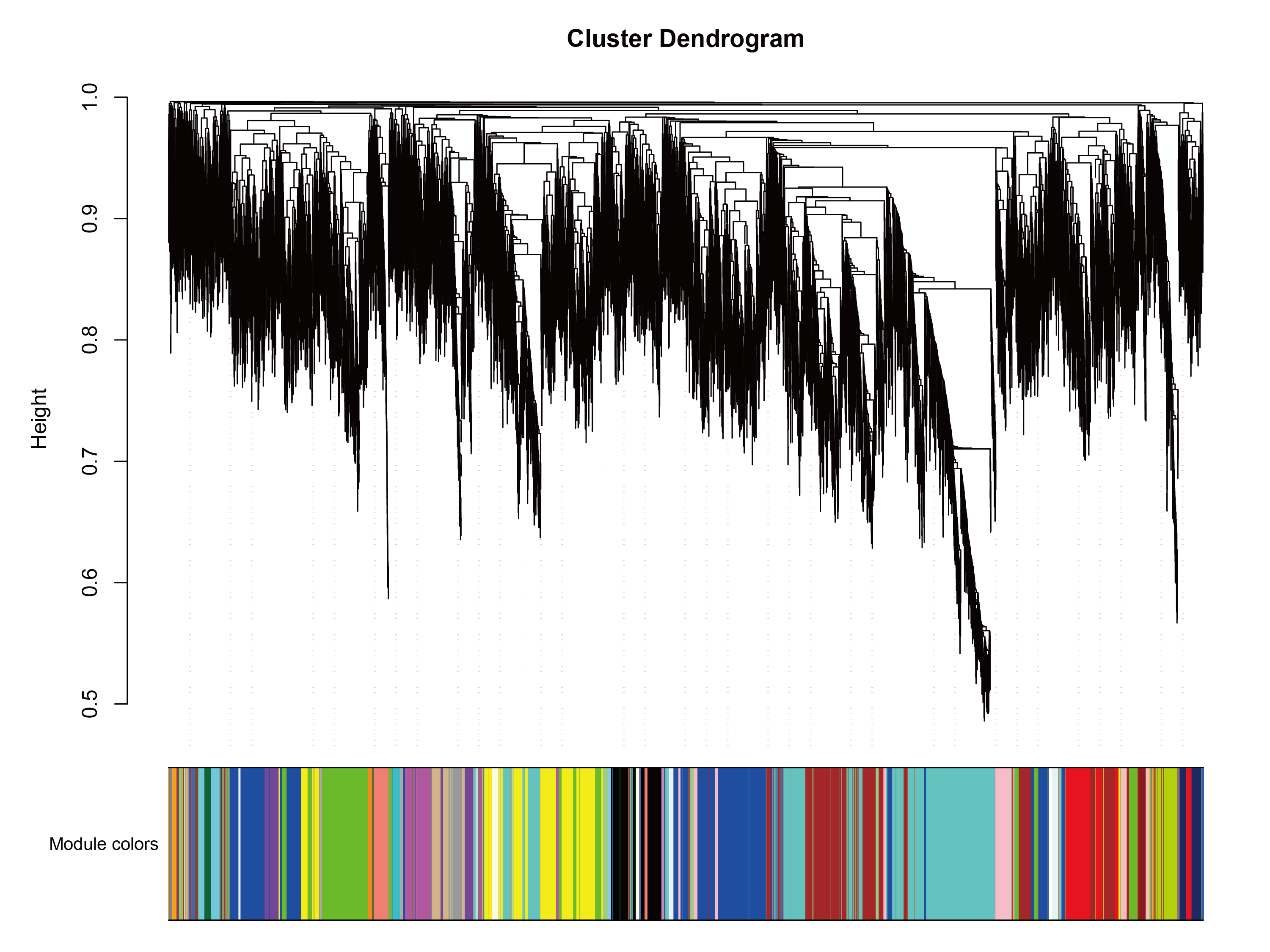


**Figure S4 Co-expression modules cluster dendrogram of samples at CRPV.** The upper part of the diagram shows the gene cluster and bottom half of figure shows the Module colors of each gene.


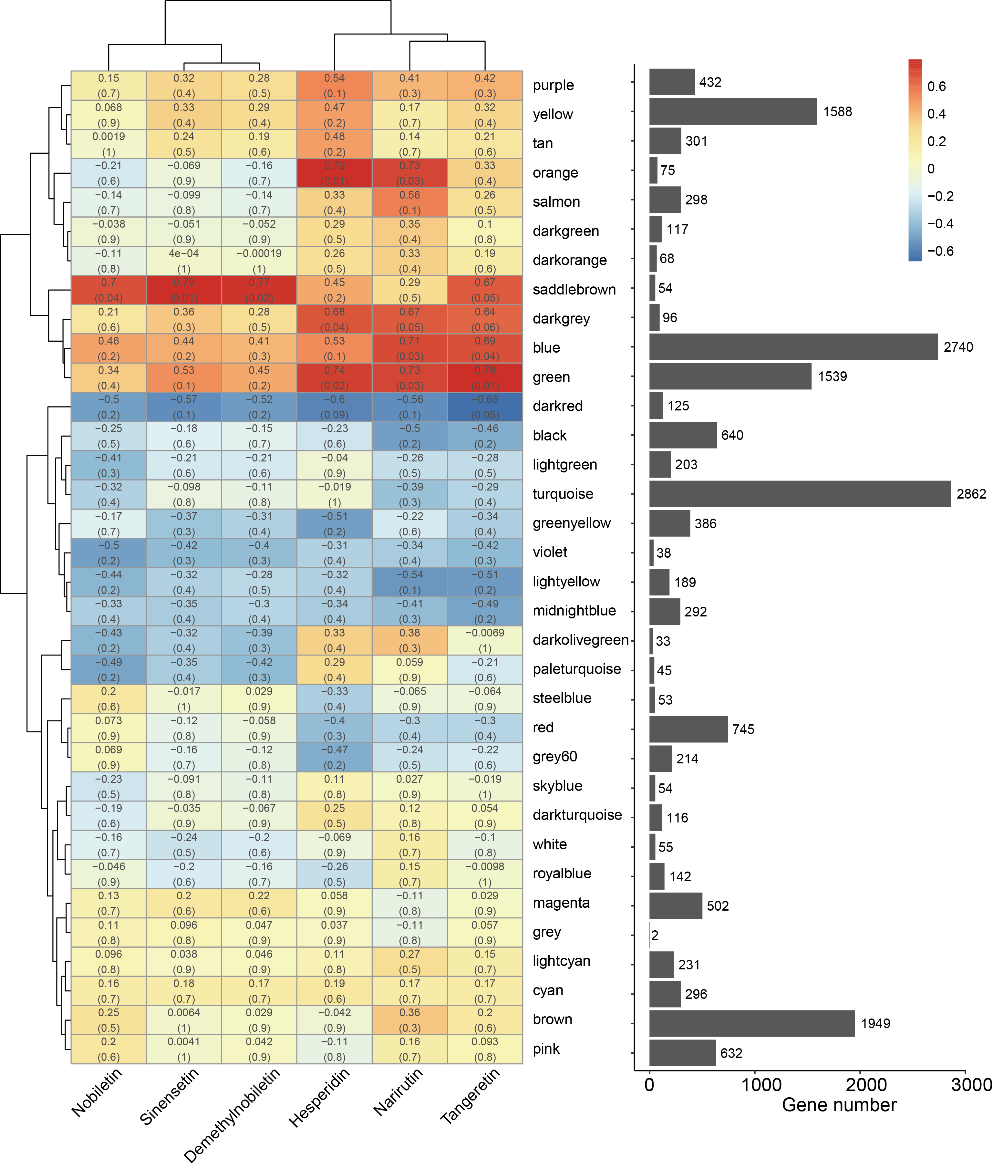


**Figure S5 WGCNA network and module–trait correlation analysis (CRPV period).** Correlations of flavonoids with WGCNA modules. Each row corresponds to a module and is labeled with the names of module. The columns correspond to flavonoids. The color of each cell indicates the correlation coefficient between the module and the flavonoids. The top number in the cell represents the correlation coefficient, and the bottom one in parentheses represents the *p* value. The bar plot on the right represents the gene number of each module.


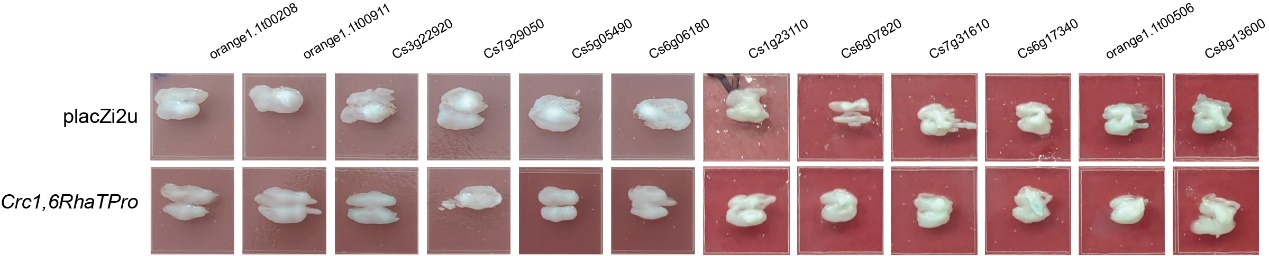


**Figure S6 Y1H analysis of *CrcMYBF1* binding to the *Crc1,6RhaT* promoter**


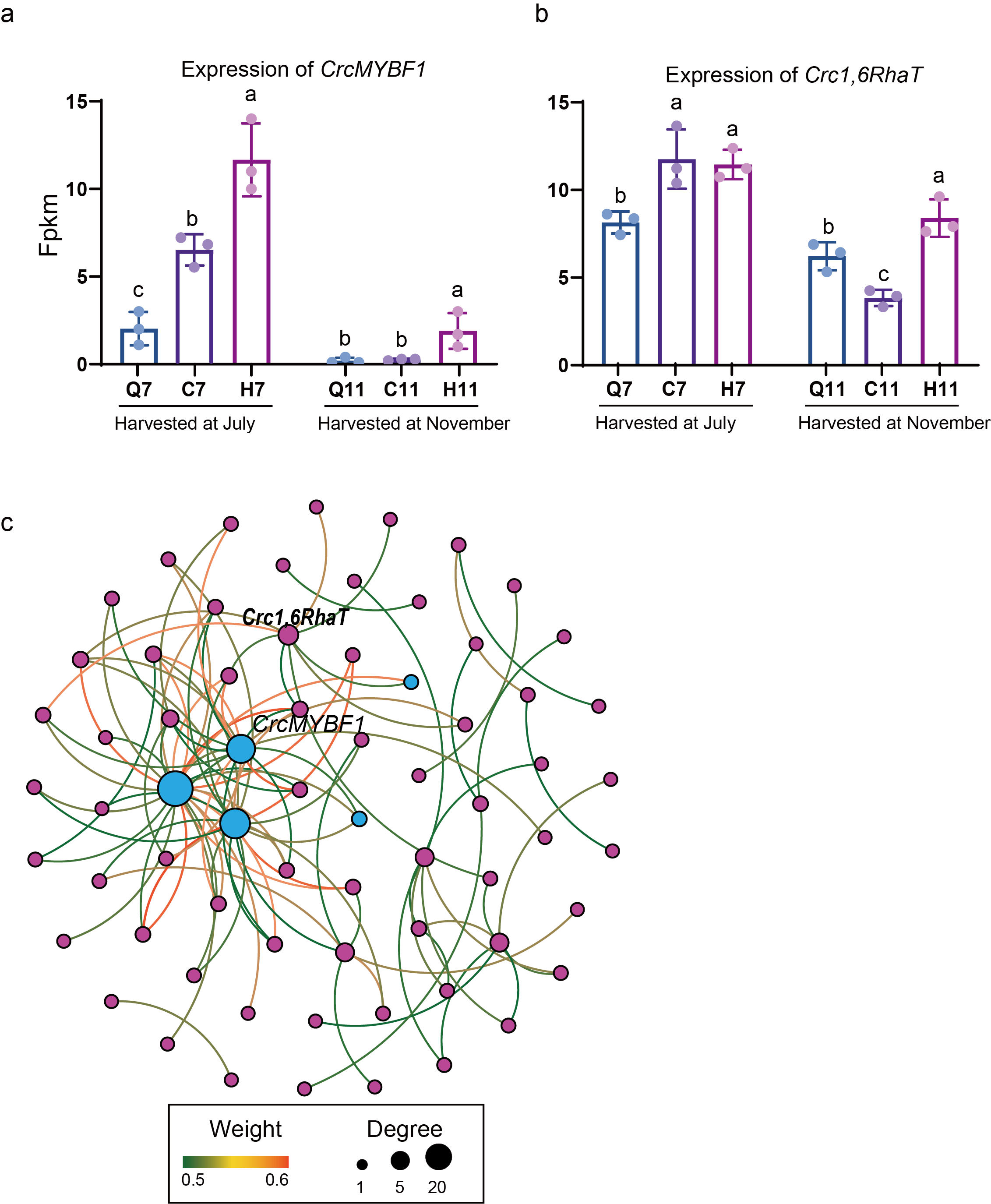


Figure S7 The expression of *CrcMYBF1 and Crc1,6RhaT*

(a, b) The expression of *CrcMYBF1 and Crc1,6RhaT.* The lower-case letters on top of the bars denote statistically different (*P* value<0.05) groups. (c) The co-expression network of hub-gene in green module. Node size represents the degree of the genes in the network.
